# Embedded Bio-Mimetic System for Functional Electrical Stimulation Controlled by Event-Driven sEMG †

**DOI:** 10.3390/s20051535

**Published:** 2020-03-10

**Authors:** Fabio Rossi, Paolo Motto Ros, Ricardo Maximiliano Rosales, Danilo Demarchi

**Affiliations:** Dipartimento di Elettronica e Telecomunicazioni (DET), Politecnico di Torino, 10129 Torino, Italy; fabio.rossi@polito.it (F.R.); paolo.mottoros@polito.it (P.M.R.); s251237@studenti.polito.it (R.M.R.)

**Keywords:** surface electromyography, event-driven, functional electrical stimulation, embedded system

## Abstract

The analysis of the surface ElectroMyoGraphic (sEMG) signal for controlling the Functional Electrical Stimulation (FES) therapy is being widely accepted as an active rehabilitation technique for the restoration of neuro-muscular disorders. Portability and real-time functionalities are major concerns, and, among others, two correlated challenges are the development of an embedded system and the implementation of lightweight signal processing approaches. In this respect, the event-driven nature of the Average Threshold Crossing (ATC) technique, considering its high correlation with the muscle force and the sparsity of its representation, could be an optimal solution. In this paper we present an embedded ATC-FES control system equipped with a multi-platform software featuring an easy-to-use Graphical User Interface (GUI). The system has been first characterized and validated by analyzing CPU and memory usage in different operating conditions, as well as measuring the system latency (fulfilling the real-time requirements with a 140 ms FES definition process). We also confirmed system effectiveness, testing it on 11 healthy subjects: The similarity between the voluntary movement and the stimulate one has been evaluated, computing the cross-correlation coefficient between the angular signals acquired during the limbs motion. We obtained high correlation values of 0.87 ± 0.07 and 0.93 ± 0.02 for the elbow flexion and knee extension exercises, respectively, proving good stimulation application in real therapy-scenarios.

## 1. Introduction

Nowadays, an increasing number of active rehabilitation techniques are moving to the bio-mimetic approach, which relies on the analysis of the surface ElectroMyoGraphy (sEMG) signal for, e.g., the application of Functional Electrical Stimulation (FES) [1], with the aim of physiologically controlling the muscle functional restoration as much as possible [2]. In particular, FES employs low energy current pulses to modulate the muscle contraction [3] where a complex stimulation pattern, useful to activate the group of muscles involved in a movement, is regulated by sEMG envelope evaluation or by muscle force indicators (e.g., Root Mean Square (RMS), Absolute Rectified Value (ARV)) [4].

In a practical application, the sEMG processing and FES control is a fundamental task to be carried out in real-time [5]. Since the run-time performance bottleneck could be easily related to the use of a general purpose computer for the FES control (often concurrently running, or loaded with, many other unrelated applications or functionalities, leading to unpredictable performances), here the idea is to replace it with a dedicated embedded system. In this regard, major concerns will be the effectiveness and safety of the stimulation and the resulting performance, i.e., a latency short enough to fulfill the real-time constraints and the quality of the stimulated movement.

We propose an embedded bio-mimetic FES system based on the Average Threshold Crossing (ATC) event-driven technique applied to the sEMG signal. The ATC essentially compares the sEMG signal with a threshold [6]: the Threshold Crossing (TC) events generate the quasi-digital TC signal, which is characterized by a digital waveform carrying analog (time-based) information. The ATC parameter is then computed by counting the number of TC events during a time window. In [7], we have demonstrated the correlation among ATC, ARV and the muscle force: in particular, having 0.95 ± 0.02 ATC-force w.r.t. 0.97 ± 0.02 ARV-force correlation, the ATC parameter can be used as indicator of muscle activity [8]. In this way, the event-driven approach enables the implementation of a low-complexity on-board feature extraction process, divided into two steps (TC generation and ATC computing), which can be directly performed in hardware [9,10], supporting, e.g., the recognition of different gestures [11,12,13,14]. While the theoretical background of ATC is quite similar to others common sEMG features, e.g., Zero-Crossing (ZC) or Wilson Amplitude (WAMP) [15], our event-based approach could overcome signal processing limitations for embedded feature extraction [16]. In particular, ZC and WAMP calculations are achieved by analyzing an already digitized signal, leading to high time, processing and power consumption, while by implementing in hardware our proposed ATC approach we are able to relax these issues.

Therefore, the minimal data size of the ATC information [10] and its sparsity (due to its event-driven nature) perfectly matches the low computational capabilities of an embedded system. Evolving from the architecture presented in previous works [10,17,18], with the aim of making the system portable and improving the run-time performance, we replaced the personal laptop, and the software based on the MATLABmathsizesmall^®^ & SIMULINKmathsizesmall^®^ environment, with a Raspberry Pi 3 B+ as the processing and control core of the system, running a multi-platform software. Its main tasks are the management of the sEMG multi-channel wireless acquisition, the computation and update of the FES parameters from the ATC data, and the safe control of the stimulator. The software features a Graphical User Interface (GUI) as well, to monitor and control every aspect of the system, eventually guiding the user into setup different and personalized stimulation sessions.

From the application point of view, typical scenario consists in the reproduction of functional movements between two subjects in the therapist-patient rehabilitation context: The muscular activity monitored from an healthy subject (therapist), e.g., doctor, physiotherapist, during the execution of a movement, is processed in order to define the FES pattern to be applied to a second subject (patient) in order to induce the replication of the same movement.

This manuscript extends what already presented in [19] by discussing the design choices and details about the system architecture, focusing both on hardware and software aspects, and the related development approaches; finally, further system characterization and validation results are presented, as well as in-vivo experiments.

The paper is organized as follows: Section 2 presents the overall system architecture and details the design and development of both the hardware and software parts; Section 3 presents the results of the validation and characterization of the developed embedded system, while Section 4 the results of in-vivo experimental tests are reported; results, with particular emphasis on the feature of the embedded system, are then discussed and compared with related works in Section 5; in the end conclusion and future perspectives are outlined in Section 6.

## 2. System Architecture: Design and Development

### 2.1. Overview

**Figure 1:**
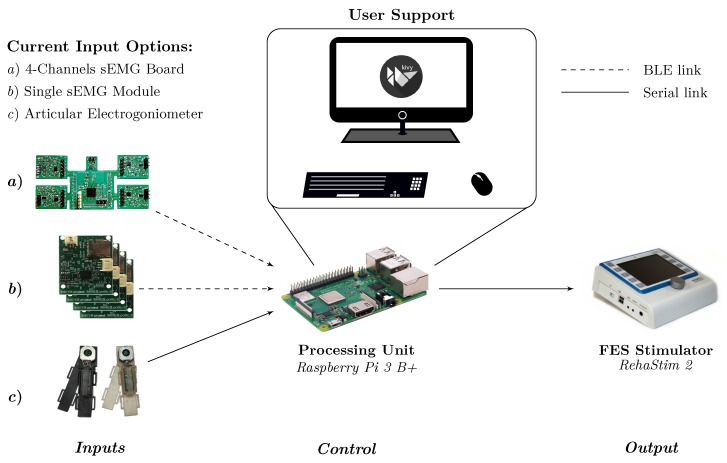
System hardware and user interface architecture: The Raspberry Pi acts as control logic linking the input devices (i.e., surface ElectroMyoGraphic (sEMG) acquisition board and electro-goniometer) with the output (Functional Electrical Stimulation (FES) stimulator).

A description of the proposed system can be conceptually schematized into inputs, control and output logical macro areas, as represented in Figure 1, according to the actions flow from signal acquisition to stimulation application. Data acquired by input devices (i.e., muscular activation and limbs motion) are processed by the control unit in order to drive the FES application through the output device.

We designed a flexible enough framework by developing a multi-platform software core, compatible with widespread Operating Systems (OSs) (such as Microsoft^®^ Windows^®^, GNU/Linux and Android), able to run on commonly available devices, i.e., PC, laptop, tablet, smartphone as well as Raspberry Pi.

Among all the possibilities, we defined our optimized embedded version of the system as Reference Hardware Setup (RHS), which comprises individual acquisition channels for sEMG and electro-goniometers as inputs, Raspberry Pi as control logic and the RehaStim 2 FES stimulator as output. With respect to RHS, other configurations are characterized by changes in the inputs and control devices (i.e., a Microsoft^®^ Windows^®^ or GNU/Linux PC), which lead to slight variations in the wireless connectivity management and software structure.

### 2.2. Hardware Platform

The input devices are the sensors useful to record the signals of interest, i.e., the electrical signals produced by the muscles contraction (sEMG signal) during the execution of a movement and the angular signals representing the limbs motion of the human body.

In the first case, the employed device has to amplify and filter the muscular signal in order to allow its interpretation, since the raw signal amplitude varies between hundreds of µV and tens of mV [20]. Therefore, referring to the guidelines reported in [21], we developed an analog conditioning circuit for the bio-signal [9], which, using the three-electrodes differential approach (two as sources, one for reference), provides 1000 gain factor in the 30 Hz to 400 Hz bandwidth (obtained as a cascade of a differential first-order high-pass filter [22] and a second-order Sallen-Key low-pass filter [23]) in order to filter out electrode-skin movement artifact and high-frequency noise. Moreover, since the sEMG module has to be coupled with FES stimulator, we added overvoltage protection diodes on the channels input. As introduced, we carried out the first step of our event-driven signal processing by extracting the TC signal using an hysteresis voltage comparator ( 30 mV) so to avoid spurious glitches. The average counts of events (ATC) is then computed at the digital interface with the microcontroller (MCU): in [10], we demonstrated how to accomplish this task minimizing the MCU resources to a GPIO interrupt, which detects the TC digital events, and a timer, which defines the observation window. The length of the window is set to 130 ms as reported in the tests presented in [9], where this value has been proved to be an optimal trade-off between the time resolution of the muscle activation and the discrimination of different levels of generated muscular force.

**Figure 2:**
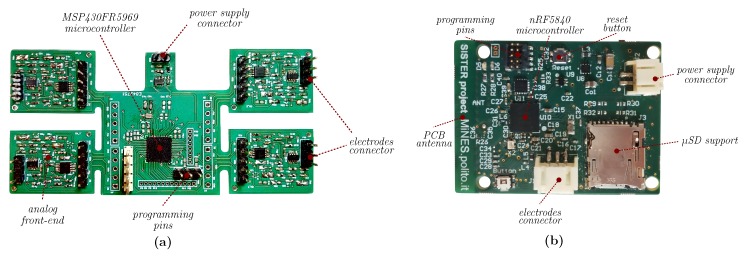
Custom sEMG acquisition device: (**a**) Four-channels board for multiple muscles monitoring, (**b**) independent single-channel module.

We propose two solutions based on this acquisition and processing architecture, shown in Figure 2, depending on the user needs: The first option (a) is a complete four-channels board suitable for multiple-muscle monitoring on the same limb, e.g., extensor and flexor muscles of human forearm, while the second one (b) is a stand-alone single-channel module to be used independently when an individual detection is advantageous, e.g., biceps- and triceps- brachii muscles during the elbow flexion and extension. We equipped both solutions with wireless connectivity in order to improve a freedom movement executions, avoiding wiring hindrance, and to make the systems fully wearable: among the wide list of wireless (standard) option, we chose the Bluetooth Low Energy (BLE) protocol (stack 4.1 [24]) because of its low-energy features, which perfectly match with battery-device requirements. In particular, we equipped (a) with the Microchips RN4020 [25] module (with its own antenna), while in (b) the same MCU used for computing the ATC runs the BLE stack and directly feeds a PCB antenna, designed referencing to [26].

**Figure 3:**
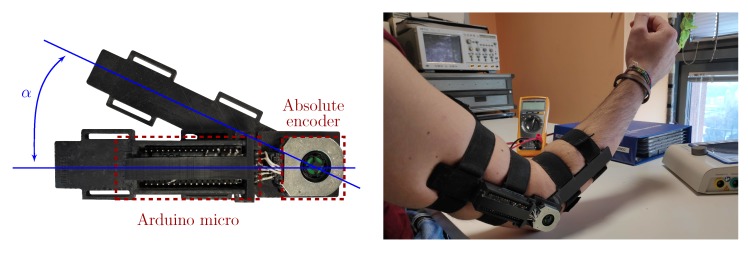
Wearable electro-goniometer developed to test the system performance and to provide an angular feedback on the ongoing stimulation.

As second input typology, we developed custom electro-goniometers in order to record the limbs motion in form of electrical signals. Figure 3 shows their structure (very similar to standard goniometer’s one), which basically consists of two parts fixed by a pivot at one extremity. Employing an absolute capacitive modular encoder, i.e., the AMT20 [27], and placing its center in correspondence of the pivot, we were able to detect the goniometer’s angle decoding the encoder shaft position related to its inner capacitance changes. Angle values are represented on 12, with a 0.2 accuracy and, since the AMT20 presents an SPI output line, we interfaced it with an Arduino micro MCU [28] in order to sample the signal at 80 Hz (appropriate w.r.t. human movement velocity [29]) and to transmit it to an external device (via USB cable) for graphical representation. The goniometer case has been manufactured by a 3D printing process, employing the Form 2 printer [30] with a bio-compatible photo-reactive resin, which allowed us to design an anatomical comfortable and lightweight structure. Four elastic strips secure the electro-goniometer in the proper location on the limb, ensuring its pivot to be in position with the rotation center of the articulation.

The control of the induced FES pulses depends on how they are electrically generated and which pulse parameters can be modified during the stimulation. We decided for the medical-certified RehaStim 2 [31] because it allowed us to have an advanced control on the pulse definition per channel and the possibility to be easy interfaced with an external device by means of the ScienceMode2 bidirectional communication protocol [32].

**Figure 4:**
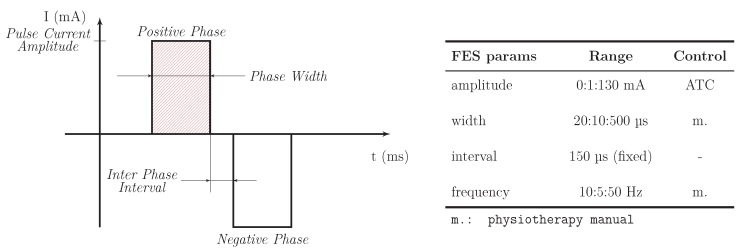
Rectangular biphasic current pulse generated by RehaStim 2 stimulator and its parameter.

The generated current pulses are characterized by a biphasic rectangular shape, shown in Figure 4, whose configurable parameters are the pulse amplitude, the stimulation frequency and the phase width, while the inter-phase interval is fixed to 150 μs guaranteeing a proper stimuli excitability [33].

Therefore, considering the ATC dependency on the muscle force (e.g., correlation between ATC and sEMG amplitude/energy indicators), our idea has been to modulate the FES pulses intensity on the basis of such parameter, while for the other settings we referred to the physiotherapy manual provided with the stimulator [34]. In this way, the modulation approach allows us to excite the muscle fibers with the proper amount of current during all the phases of a movement session (warm up, increasing force, relaxation as well as resting state) and for a wide list of exercises.

Last part of the system is represented by the Raspberry Pi, model 3 B+ [35], working as control logic which manages the entire system. Indeed, it runs the main software controlling the data acquisition, its processing, the stimulation definition and application. Moreover, since this Raspberry Pi is equipped with four USB ports and a full size HDMI, we improved the system usability developing a complete GUI and employing some peripherals, as keyboard, monitor and mouse.

As discussed in Section 2.1, different devices can act as control unit appropriately configuring the hardware: As an example, if a Microsoft^®^ Windows^®^ OS PC is used as control logic, the CC2540-Dongle [36] module is needed to communicate with the acquisition devices (limiting the maximum number of simultaneously connections to three) since Windows^®^ machines do not allow an easy access to the Bluetooth interface.

### 2.3. Software Overview

As previously introduced in Section 2.1, although the project is finalized to the development of an embedded system, we want to provide a modular and flexible software core able to fulfill the compatibility requirements of different OSs. As previously introduced in Section 2.1, we provided a modular and flexible software core able to fulfill the compatibility requirements of different OSs. Consequently, from the development standpoint, we based the software on the Python language, because of its cross-platform nature, its widespread adoption, and the large availability of third-party multi-platform libraries (such as standard library for multi-threading features or Kivy library [37] for the GUI). Moreover, the embedded software has been based on an object-oriented (OO) framework in order to promote flexibility, modularity and robustness [38] (e.g., leveraging encapsulation, inheritance, and composition features), allowing a seamless integration and management of several devices (e.g., different input modules) along with the possibility of future integration of new processing algorithms. We also implemented a multi-threaded architecture in order to map the functional tasks onto different running threads [39], so to optimize the use of computational resources and to avoid complex (run-time) code interdependencies.

#### 2.3.1. Classes Diagram Overview

**Figure 5:**
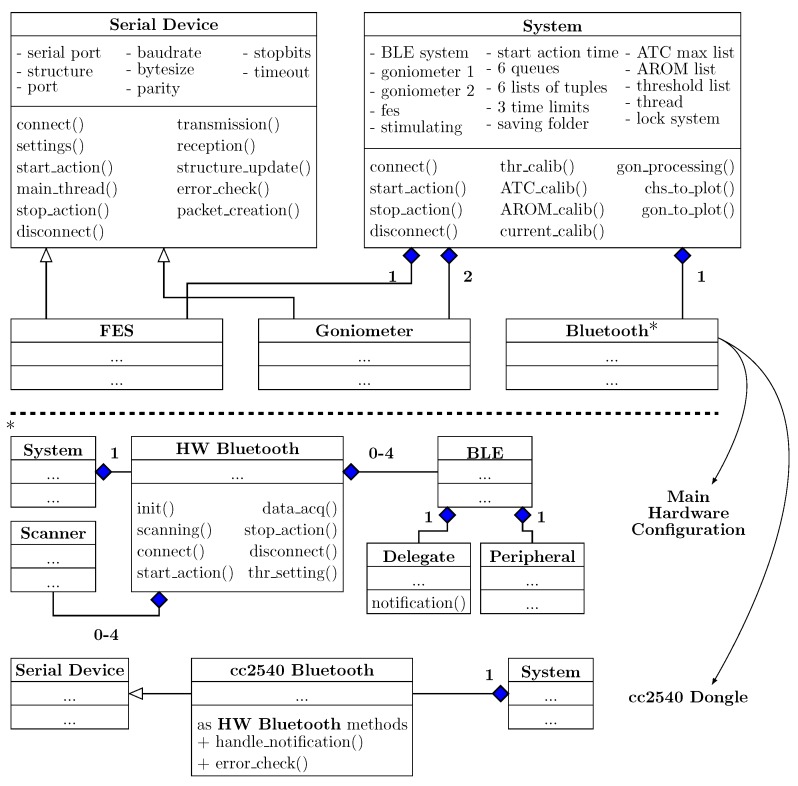
Classes diagram (UML) of our OO software organization. Bluetooth class implementation depends on system configurations.

As shown in the Unified Modeling Language (UML) diagram in Figure 5, the main System object is composed by four sub-objects: The FES class representing the stimulator, two Goniometer classes for the developed electro-goniometers and a Bluetooth class, which can have different implementations depending on the hardware configuration.

Since both the goniometers and the stimulator are wired connected to the control unit, their classes inherit from an abstract custom Serial Device class, which provides a standard interface for every serial device (i.e., serial port, baudrate, stopbits, etc. attributes or connect(), settings(), transmission() methods and so on). Specific methods of different serial devices have been overwritten in order to provide the proper interfacing with the control unit.

Regarding the BLE software, it depends by system configurations: if the RHS is used, we combined the BlueZ [40] Linux Bluetooth stack with the bluepy [41] Python library (specific for low energy features). In particular, the HW Bluetooth class is composed by a variable number (zero to four) of BLE connections, which in turns consists of by a Delegate (notification data handling) and a Peripheral (bluepy instance for encapsulating BLE BlueZ connection) objects, and a Scanner, which seeks for advertising devices. On the other hand, if a common PC is employed, the CC2540-Dongle module is needed and, since it communicates through a serial port with the workstation, the Bluetooth class inherits from the Serial Device one.

#### 2.3.2. Multi-Threading

**Figure 6:**
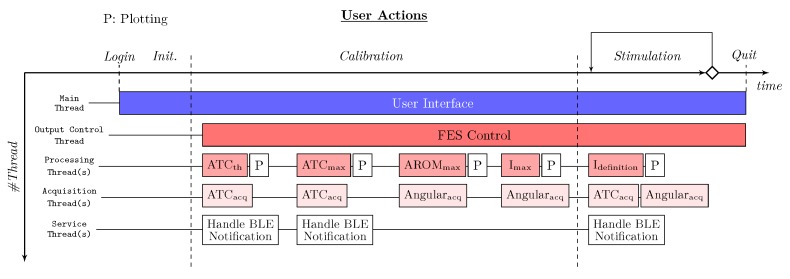
Multi-threading structure during a typical stimulation scenario.

Figure 6 shows the multi-threading structure of the system and the running state of the involved threads during a typical stimulation session.

The Main Thread starts after the user login and runs all along the session waiting for the user inputs, at which correspond the creation of child threads, handling the user interface. As primary sub-thread, the Output Control Thread manages the communication with the stimulator, e.g., watchdog timer, packet creation etc., during the calibration and stimulation phases. Moreover, the Main Thread runs all the calibration-step threads (i.e., ATC_th_, ATC_max_, AROM_max_ and I_max_, details in Section 2.4) during the settings and the I_definition_ threads when the stimulation is applied, globally defined as Processing Threads. Each of them is also supported by a Plot thread, represented by white rectangle, which graphically represents the useful signals. Finally, we developed the Acquisition Threads, divided into ATC_acq_ and Angular_acq_ for the ATC and angular values acquisition, and Service Thread for BLE notifications managing. Data exchange among threads is organized with queue objects; therefore, each thread implements a specific method in order to continuously check the queue status.

#### 2.3.3. Graphical User Interface

The GUI has been developed choosing the Kivy Python library [37], due to OS inter-compatibility, modern layout, open-access feature and optimized performance [42], in order to have an easy, intuitive, and practical high-level control of the application.

**Figure 7:**
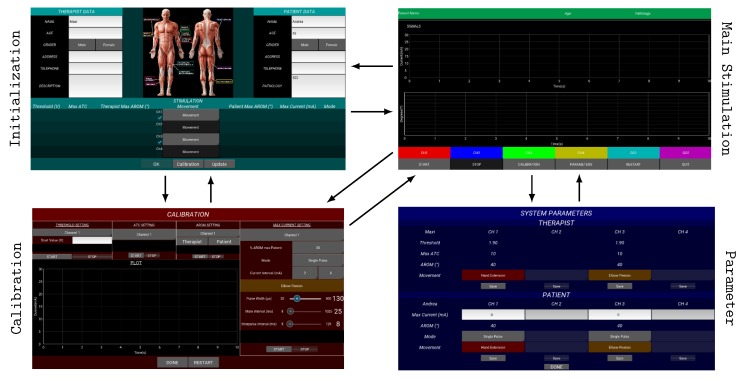
Kivy main screens: The Initialization one allows the user to store subjects information and set up the system; in the Calibration one the calibration process is achieved, and the optimized parameters can be visualized, saved and modified into the Parameter screen; finally, the Main Stimulation screen runs the stimulation and graphically represents the signals of interest. Arrows highlight transitions among screens.

Figure 7 shows the main four screens of the GUI. In the Initialization one, the user inserts the personal information of therapist and patient, and chooses the system configuration (acquisition and stimulation channels) along with the movement that will be executed. The Calibration screen is properly designed to perform the calibration process, whereby the acquisition and stimulation parameters are optimized for the user-case. Subjects data are used to build up a database, useful to fast-configure application settings avoiding the calibration steps. In the Main Stimulation screen, the stimulation can be started and stopped, and the useful signals are graphically represented (i.e., pulse amplitude and angular signals) in order to provide a visual feedback for the therapist. Lastly, the Parameters screen allows the user to modify the parameters or save them if multi-session scenarios are expected. Transitions among the screens, represented by black arrows in Figure 7, have been arranged using the Screen Manager object, facilitating user navigation among sections.

From an OO prospective, all the screens directly inherit from the Kivy Screen class, with the exceptions of the ones containing graphs (i.e., Main Stimulation and Calibration) which are also defined by the MyPlotScreen class since it possesses Kivy plotting objects. Thus, the System is aggregated in every main screen where the system actions run through screens widgets.

Lastly, on the Raspberry Pi, we changed the RAM memory assigned to the Graphics Processing Unit (GPU) from 64 MB to 256 MB in order to execute the GUI without impacting on the graphical resources.

### 2.4. ATC Dataflow: Processing and Calibration

The definition of the FES pulses amplitude dependent on the ATC values is the core of the FES control mechanism, linking the data acquisition with the stimulation one. Since embedded device has extremely low-computational power, we needed to implement this process trying to maintain the complexity lower as much as possible, also considering fast computing approach to respect real-time requirements. In this scenario, taking advantage of the sEMG-ATC (pre-)processing, our idea is to mimic the simplicity of a look-up table structure: Basically, we organized it as two matrices architecture, one for the ATC values and one for the FES current ones, with one-to-one cell correspondence between them.

**Figure 8:**
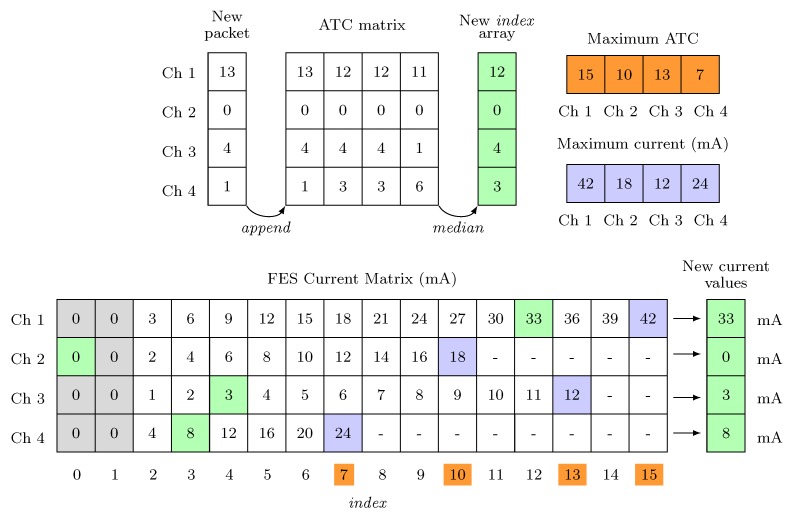
Average Threshold Crossing (ATC)-FES definition process: Green cells represents the links between inputs ATC data and outputs pulses current; orange labels identify the FES current Matrix indexes defined by the Maximal ATC calibration step; blue values correspond to the maximal FES current calibrated with the Current limitation process.

Typical application scenario, considering *n* active channels, is represented in Figure 8: Every time a new BLE packet arrives, containing the ATC data of n channels, the received data are appended to an n×4 matrix (ATC matrix), which also includes the three past ATC-window data. Then, the row-median operation is computed in order to obtain a robust ATC value without any noise corruptions. Since the ATC matrix is continuously updated (every ATC window), this operation basically represents a moving median. In this way, we obtain a n×1 array, whose values are interpreted as indexes pointing to the FES current values stored into the FES Current Matrix. Once the new stimulation data are defined through this algorithm, a FES data packet is built up and the command is transmitted to the stimulator.

However, since different subjects could produce different ATC values or be stimulated by a diverse amount of current, a calibration process for the optimization of the acquisition (therapist) and stimulation (patient) parameters is fundamental, permitting us to develop a flexible system, able to suit different users, while maintaining the benefits of a proper and safe per-subject stimulation. Hence, we defined a four-steps calibration process as follows:

1. Threshold setting: The generation of the TC events strongly depends on the threshold value. Therefore, we tried to optimized the TCs setting the threshold just above the sEMG signal baseline in order to maximize the events with the minimal muscle effort. To accomplish this task, the therapist has to maintain a rest limb condition and, starting from an initial threshold value, we decrease it step-by-step until we find the baseline. Final threshold is set 30 mV above baseline reflecting voltage hysteresis comparator behavior.
2. Maximal ATC: The therapist has to repeat the movement to be calibrated at least four times. The maximal ATC value produced by the subject is calculated as the median value among the maximum of each repetition. This value limits the index dimension of the array, related to the calibrated channel, inside the FES Current Matrix, highlighted in orange in Figure 8.
3. AROM evaluation (optional): The maximal Absolute Range of Motion (AROM) of the involved articulation has been computed by processing the angular data of both therapist and patient. This measure standardizes the FES application and provides a comparison feedback between the voluntary movement and the stimulated one. We defined it as an optional step since the use of the electro-goniometers is not mandatory.
4. Current limitation: We define the maximal current, useful to properly reproduce the movement, as the 110% of the current able to produce a 30% AROM variation in the stimulated subject. If the goniometer is not used, this step can be visually performed. Maximal Current values, represented in blue in Figure 8, related to the indexes defined by the Maximal ATC, define the proper stimulation values inter-step.

Following this approach, we are able to set up our structure with a perfect matching between the muscular activation of the therapist and the pulses amplitude to adequately stimulate the patient limb. Looking at the example represented in Figure 8, the FES Current Matrix has a different column-dimension for each channel defined by the Maximum ATC values. In this way, setting the Maximum current values, we are able to define step and range of pulses amplitude. Concluding, simply controlling the lower values of the stimulation matrix (FES Current Matrix[(:, 1:2)], grey cells), combined with the moving median gate operation, we are able to implement a very low complex but efficient noise-gateway control.

## 3. System Validation and Characterization

The performances of the control unit have been studied by analyzing the real-time FES control processing, fundamental to achieve the proper online modulation of pulses amplitude, and examining how the developed software impacts the workstation resources, so evaluating memory, graphic and computational cost. Due to the multi-platform nature of our software, we carried out these tests comparing its behavior running on two different control units: in particular, we employed the Raspberry Pi 3 B+, equipped with a Cortex-A53 (ARMv8) 64 bit, running at 1 GHz, 1 GB RAM and Raspbian OS, to evaluate the performance of the embedded version; conversely, a Toshiba Satellite L830-14J PC, equipped with an Intel Core i3-3227U with 1.9 GHz clock frequency, 4 GB RAM and Microsoft^®^ Windows^®^ 10 OS, has been used to simulate personal laptop application scenario.

### 3.1. Latency Measurement

As mentioned in Section 2.1, the system can adopt different architectures depending on which sEMG acquisition device is used and by the employed processing unit. As a consequence, we defined five hardware configurations (**Cx**) to be tested, listed in Table 1. The latency has been evaluated for two crucial sections of the application: The FES current definition, which concerns the definition of the new pulses amplitude on the basis of the latest ATC values, and the Plotting, which regards the representation of both the angular signals and the FES currents over time. The duration of the test has been set to 3 min in order to obtain sufficient values (180 s/$ATC_{window}$ = 180 s/ 0.13 s≃ 1385 measures) able to represent the system performance from the stimulation initialization to the stable working condition.

#### 3.1.1. FES Current Definition

This method represents the logical core that links the ATC values, describing the muscular activity, to the FES current values, which specify the amplitude of the incoming pulses. As described in Section 2.4, we implemented this functionality using a lookup table structure in order to minimize the complexity as well as the processing time. Indeed, the real-time FES definition is a fundamental task for a proper stimulation, avoiding any delay caused by data-queueing; in particular, our time constraint is directly related to the ATC window, which defines the time interval between two ATC values, and so the FES current definition processing time has to be lower than 130 ms. We tested this process studying how different methods split the workload among them and evaluating the total processing time. As details of the first test, the FES current definition is divided into five consequential methods: queue continuously checks the incoming of the new ATC values; when they are available, we append them to the ATC matrix and the median operation is performed. Then the FES_start method builds the FES packet to be transmitted to the stimulator, and in the meanwhile the plot thread is called for the graphical representation of the signals.

Table 2 reports the time profiling of the workload breakdown: As it can be observed, the majority of the time (around 90% in **C1**, **C2** and **C3**, and 97% for **C4** and **C5**) is spent inside the queue method waiting for the arrival of new ATC values, while the other sub-functions runs for a very short time. Therefore, from a methods breakdown point of view, this behavior confirms that the application works as expected, avoiding any queue formations caused by low computational processing.

**Figure 9:**
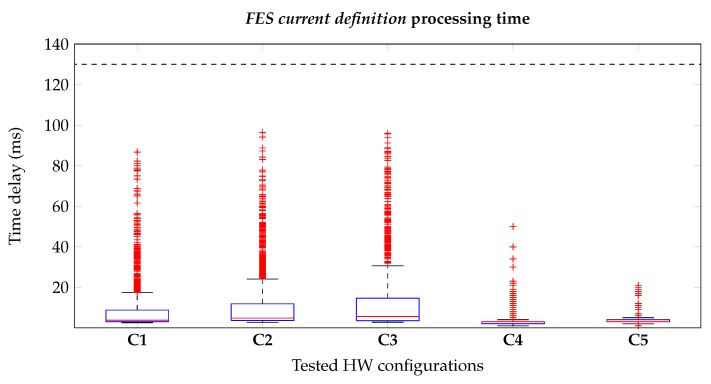
Box plots representing the time delays related to the FES current definition method for the different system configurations. Real-time constraints are respected, considering our time limitation of 130 ms (dashed black line), both in GNU/Linux Raspberry (**C1**, **C2** and **C3**) and Microsoft^®^ Windows^®^ PC (**C4** and **C5**) cases.

On the other hand, the real-time FES definition has been proved by looking at the delay data represented in the box plots in Figure 9. All the cases largely fulfill our time constraint, also considering the outliers, since none of the values is greater than 100 ms. In particular, in the Raspberry cases and PC ones the median values are below of 10 and 5 ms respectively, which avoid any possible delay between acquisition and stimulation caused by our FES definition processing. However, comparing the two OSs, laptop performances are considerably superior with respect the Raspberry ones since both hardware and software resources differ between the two architectures.

In conclusion, these results prove the low complexity implementation of our event-driven ATC-FES definition approach: considering the computational lightness of the ATC processing, based on simple mathematical relations (such as matrix and median operations) applied to a minimal data size, we were able to reach very fast pulses updates (lower than 10 ms) along with online modulation of FES parameters.

#### 3.1.2. Plotting

**Figure 10:**
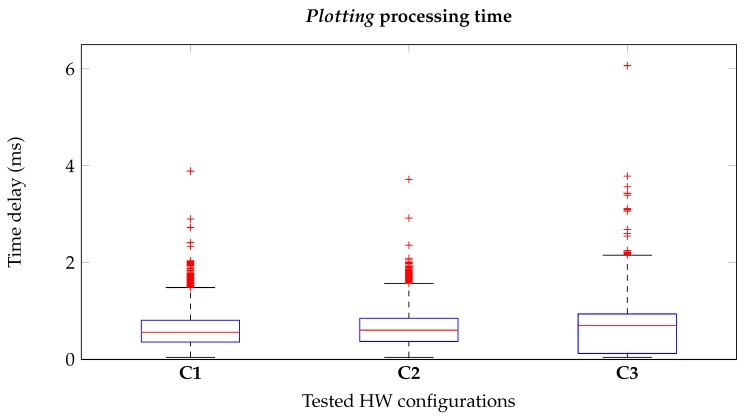
Plotting delay for the configurations employing the Raspberry Pi (**C1**, **C2** and **C3**). The very low plotting-time proves the real-time graphical representation by our application.

The above measurements are repeated for the Plotting process in order to study if the graphical representation of the signals of interest can affect the run-time performances. The plotting is based on a clock object, whose methods are the get_value and the sleep: The former gets the new ATC and angular data, and represents them on the graphs; the latter puts the object into an idle state until new data are available. As in the previous test, this analysis allows us to detect the queues formation during the plotting process, but, looking at the Table 3, we can assume this critical condition has not been reached since the 99% of the time is spent in the clock sleep state. Moreover, we measured the time spent by the Plotting thread in order to verify the correctness of the data representation, discarding the possibility to misinterpret the stimulation visual feedback. This time, we report in Figure 10 our results only for the embedded configurations as worst case scenario: Again, since the 95% of the values (IQR) are lower than 2 ms for all the cases, we can assume the real-time behavior of our plotting method.

### 3.2. Computational Performance

The information about the usage of resources at the processing side, such as the Central Processing Unit (CPU) and the Random Access Memory (RAM), is of crucial importance for the evaluation of application fluency, performance and usability. From this perspective, we carried out this test using the RHS (**C2**), considering it as the worst case scenario in terms of processing power. Hence, we monitored the system processes through the htop GNU/Linux tool, running the system without the Raspbian GUI activated in order to take in consideration only the application and all its dependencies (i.e., BLE and Kivy).

Table 4 reports the results when four channels stimulation mode has been selected: As it can be observed, the most challenging CPU performance is reached in the main stimulation procedure, where the highest amount of threads are active. Therefore, focusing on the Stimulation stage, we repeated the measures studying two different but dependent cases: first, we varied the channels number from one to four looking at resources usage changes; second, with the same purpose, we analyzed our ATC-FES current definition process both in the standard situation (i.e., append and median methods), and when a direct equivalence between ATC and current values is performed (no data processing). From the results listed in Table 5, we can see that employing one single channel we are able to reduce the CPU usage of approximately the 20% w.r.t. the complete channels configuration, and it entirely depends on the fewer or higher number of co-running threads in the two situations. Instead, as it can be observed by the two main columns, the FES current definition implementation does not affect the CPU usage, which further proves the lightweight computational cost of our approach.

On the other hand, the dynamic memory suffers just low variations among the application stages and between one or four channel cases. This behavior is mainly due by the different amount and types of widgets the GUI owns, which are directly related to the number of active channels. Hence, since there is not any differences between single or four channels GUIs, the RAM consumption is almost constant (see Table 5).

## 4. In Vivo Experimental Tests and Results

As the system is intended for rehabilitative sessions, some tests have been carried out in order to prove the correctness and appropriateness of our approach in the control of the FES application. As introduced, typical scenario considers a first subject which performs an useful movement and whose muscle activity is monitored (therapist), and a second subject which replicates the movement as consequence of the stimulation application (patient). We studied the similarity between the two movements by analyzing the limb motion signals, acquired using the developed electro-goniometers which are worn from both the therapist and patient. We compared the angular signals calculating the maximum of the normalized cross-correlation coefficient (σ) as reported in the following formula:

(1) $$\sigma =max\left(\sigma_{th\_pt,coeff}\left(m\right)\right)=\frac{1}{\sigma_{th\_th}\left(0\right)*\sigma_{pt\_pt}\left(0\right)}*\sigma_{th\_pt}\left(m\right)$$

where *m* is the lag between the signals (th, pt), and the autocorrelation product normalization limits σ values to 1 (perfect match between signals) and –1 (complete opposite signals).

We enrolled 11 healthy subjects, whose have been submitted their informed consent for our testing protocol (approved by the Bio-ethical Committee of the Università degli Studi di Torino, Italy), and we divided them into therapist-patient couples. In the next sections, we introduce the adopted methodologies for choosing electrode type and for preparing the subjects skin, and we report our complete results for upper limb exercise and the preliminary one for the lower limb exercise.

### 4.1. Electrodes and Skin Preparation

A proper treatment of the electrode-skin interface is essential in order to enhance the signal acquisition quality. Therefore, cleaning the skin surface with medical alcohol allows a removal of fat, dust and dead cell, and an increasing of the conductivity through the electrode [43]. We chose the Kendall^™^ Covidien H124G ∅24 mm [44] for the sEMG signal acquisition due to the Ag/AgCl sensor, pre-gelled surface and long-term stability. Instead, for the stimulation, we employed the 5 cm × 9 cm RehaTrode [45] rectangular self-adhesive electrodes produced by the Hasomed^®^, which are perfectly designed to be coupled with the RehaStim 2 stimulator. The main difference between these two types regards the working area, having the acquisition electrodes a higher spatial resolution while the stimulation ones cover a bigger surface to properly induce the stimulation.

### 4.2. Upper Limb: Elbow Flexion

**Figure 11:**
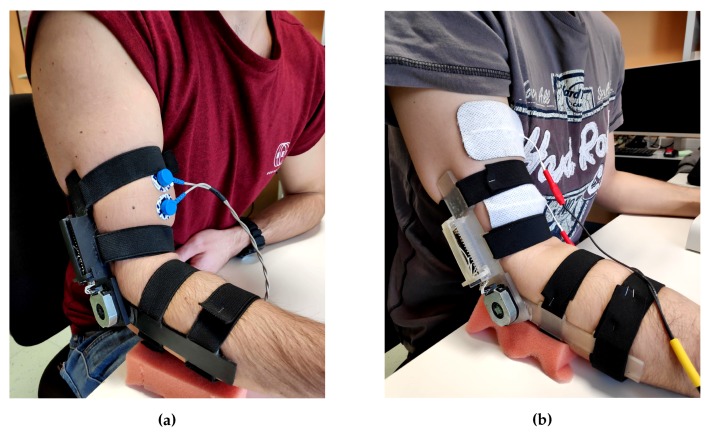
Acquisition (**a**) and stimulation (**b**) electrodes location, on the biceps femoris muscle, for the EF exercise.

As upper limb benchmark exercise, we chose the Elbow Flexion (EF) movement, which consists in the forearm motion toward the upper arm rotating around the elbow join center. The active muscles of the arm are the brachialis, which attaches the humerus to the ulna, the brachioradialis, that connects humerus and radio, and the biceps brachii, which links the shoulder blade to the radius [46]. Since our idea was to perform this first tests with minimal complexity, we decided to monitor and to stimulate only the biceps brachii also due to its accessibility by surface electrodes. Therefore, we placed the couple of acquisition electrodes at 1/3 of the line between the fossa cubit and the medial acromion, with 20 mm inter-electrode distance, and the reference one on the back of the hand, as electric-neutral area [43]. In contrast, the FES electrodes position slightly differs from the previous ones, being located one on muscle belly and the other one closer to the crease of the elbow [47], in order to have a correct muscle fibers contraction. The experimental setup is shown in Figure 11a,b.

Ten healthy subjects (five males and five females, 24–27 years old) took part to the testing phase: We divided them into five therapist-patient couples and, after the calibration of the acquisition and FES parameters, we asked them to repeat the EF exercise twelve times for each couple. A single repetition has to follow this flow: The starting position for both the therapist and patient is upright sitting, with their forearms and hands completely lean against the table, forming a 90° angle with the upper arm; then, the therapist performs the movement reaching her/him AROM, and finally returns to the starting position; once also the patient has finished the exercise, a short pause of at least 10 s prevents any muscle fatigue effects.

ATC, FES current, therapist and patient angular values have been collected during the entire session. They are successively processed in the MATLAB^®^ environment in order to extract the useful information for the comparison between the voluntary and stimulated movement. The angular signal processing consists of the following steps:

1. Segmentation of the complete signals into single epochs representing one repetition.
2. Baseline removal, since it could be different depending on the limb starting position.
3. Signals normalization to the related AROM values.
4. Computing of the maximum of the cross-correlation coefficient for each epoch.

**Figure 12:**
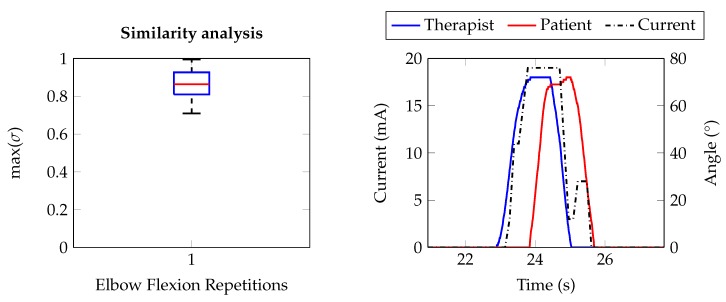
(**left**) Example of the stimulation application of a repetition of the Elbow Flexion (EF) movement: Blue and red are the angular signals of the therapist and patient, respectively; the dashed black line represents the FES current injected through the electrodes. (**right**) Similarity analysis using the maximum of the cross-correlation coefficient to compare the limb motion angular signals of the therapist and patient for the EF movement.

The box plot on the left of Figure 12 represents the entire dataset of σ values (60 measures: Five couples per 12 repetitions each one) extracted during our test campaign. As it can be observed, the distribution is Q_3_-skewered to the unity, which indicates a good reproduction of the movement, further confirmed by a median value above 0.8. Indeed, looking at the angular signals on the right graph of Figure 12, representing a single repetition, we can see how much the limb motion is similar between therapist and patient. Moreover, this graph also shows the on-line modulation of the stimulation current when the ATC values, directly proportional to the therapist limb angle, trigger the increasing, decreasing or plateau current phases. It is possible to notice that the total delay between the two movements is due to a first short processing phase, visible as distance between non-zero therapist angle and non-zero FES current, and a physiological longer one, distance between non-zero FES current and non-zero patient angle, which depends on the muscle mass and fibers contractions.

### 4.3. Lower Limb: Knee Extension

We also tried to replicate the Knee Extension (KE) movement, due to its largely employment as physiotherapy exercise. From a sitting initial position, the contraction of the quadriceps femoris muscle allows the extension of the leg with respect to the knee joint. This muscle is composed by four separate muscles: The rectus femoris, in the middle of the thigh; the vastus lateralis located on the lateral side of the femur; the vastus medialis on the medial side; and the vastus intermedius under the rectus femoris [48].

**Figure 13:**
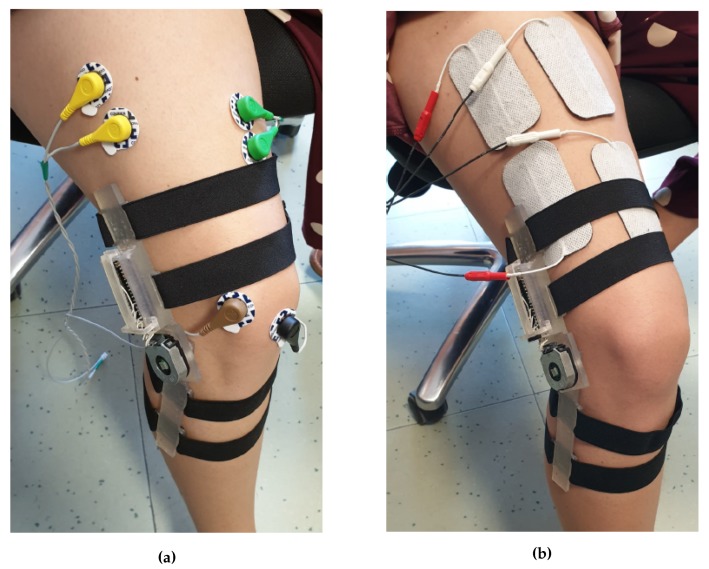
Knee extension exercise. (**a**) sEMG acquisition electrodes on the vastus lateralis and vastus medialis muscles. (**b**) the electrodes are directly placed on the muscle bellies of the vastus lateralis and vastus medialis. This locations and the electrodes dimension also contract the rectus femoris muscle improving the stimulation effectiveness.

In our test, we decided to monitor the vastus lateralis and vastus medialis, setting up a two-channel stimulation layout: in the first case, the sEMG electrodes were placed at the 80% of the line from the anterior superior iliac spine and the medial side of the platella, while in the second case, they were put at 2/3 of the line connecting the anterior superior iliac spine with the superior lateral side of the patella [43], as shown in Figure 13a. Both reference electrodes were located on the patella. On the other hand, as reported in Figure 13b, the stimulation electrodes were placed along the muscle bellies in order to cover a surface including both the muscles and the rectus femoris.

Our preliminary results consist in 13 repetitions of the movement, performed by a single female subject (24 years old). As in the EF case, both the therapist and patient need to start from an initial position, which we defined as 135° between thigh and calf. Then, the therapist extends its leg until her/him AROM and, once the stimulation is completed, at least 10 s have to be waited before next repetition.

**Figure 14:**
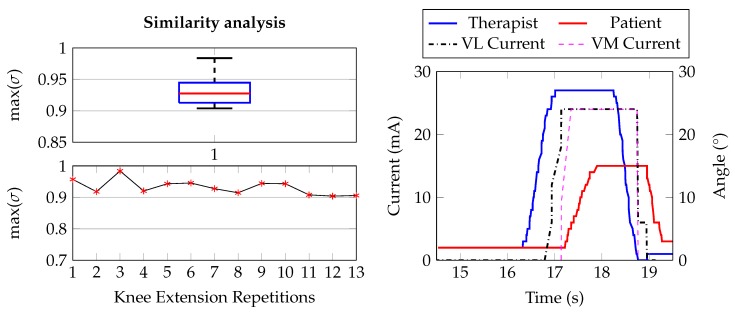
(**left**) Single repetition example showing the similarity between the two angular signals with respect to the vastus lateralis (VL) and vastus medialis (VM) stimulation currents. (**right**) Maximum of the cross-correlation coefficient for the 13 repetitions of the knee extension movement.

The cross-correlation results, calculated with the same method of the EF case, are reported on the box plot in Figure 14 (top left). However, these values are also represented by a time-graph (bottom left) in order to avoid any misinterpretations due to the low number of measures. Looking at the graphs, some considerations can be made: first of all, also for the KE movement, we obtained satisfactory results in term of similarity between the two signals, proved by the σ values completely equal or greater to 0.9. Indeed, considering the repetition example on the left graph, we can observe the similar morphology among the therapist motion and the patient one. Anyway, the maximal angular value reached by the patient is lower than the therapist one. This behavior is possibly related to the muscle physiology activation and different fibers recruitment between voluntary and stimulated contraction. One cause could be associated to the stimulation of a healthy subject, with a normal muscles condition, that, by applying large values of stimulating current, could lead to sense of pain. Hence, we limited the current to the values represented by the dashed line in the graph avoiding this situation. A second possibility could be related to which muscles have been stimulated: A complete leg extension involves the total contraction of the quadriceps, while superficial electrodes could not result in the proper shortening of the deeper fibers, consequently producing a limited movement. Anyway, further studies will allow us to set up more complex stimulation scenarios, which will induce a better reproduction of the movement.

## 5. Discussion: sEMG-FES Systems Comparison

Table 6 reports a summary of literature works in the field of FES application triggered by sEMG signal analysis. The classification includes which control feature (e.g., RMS, envelope, ATC) has been used to online modulate one or more FES parameters, as well as the employed hardware, which summarizes the processing capability and the possibility to transfer the FES algorithm into an embedded device. Moreover, these systems could be also analyzed by considering additional features such as wireless connectivity, modularity and number of active channels which foster system wearability, future sensors and algorithms integration, and application typology. Lastly, since real-time behavior remains a major constraint, rightmost column shows the latency (FES pulses update period) measurements calculated as FES processing delay or (whenever available) the therapist-patient delay.

A complete comparison between our system and those reported here is not straightforward due to the large variety of analyzed features; anyway, some considerations about different methods and performance can be carried out. In [51] authors used the threshold crossing feature extraction to modulate the stimulation frequency of the FES pulses, achieving a very promising FES definition latency of 142 ms, directly comparable to our outcomes. However, since the sEMG processing has been embedded in the MCU, a standard sampling approach is needed, which includes peripherals management and relative expensive processing power; on the other hand, with our event-based approach, MCU resources could be drastically reduced by implementing ATC in hardware. At the same time, linking the TC events with the stimulation frequency results particularly interesting in order to reproduce motor fibers firing rate; in our system we preferred, as a first step, to modulate the intensity, but a frequency-control approach could be easily implemented thanks to the flexible and modular architecture of our system. Another interesting frequency modulation has been presented in [54], which evaluates the sEMG entropy on an MCU architecture, but limiting the number of controlled channels to one.

With reference to the pulse amplitude control, it could be performed by extracting different features from the muscle signal (e.g., RMS [49], envelope [50] and force [52]) or using data-fusion techniques with different type of sensor (as Inertial Measurement Unit, IMU [53]): While the final latency among these works and our proposed system is quite similar, and respects the real-time constraints for such type of application, the processing methodology does not always allow to use an MCU [53] and, where it is possible, raw data acquisition seems to be the common adopted solution.

As another comparison point, to the best of our knowledge for this application, our architecture is the only one which presents a modular system structure, thanks to the combination of chosen programming language and strategies.

Lastly, looking at the ATC-FES system evolution from previous versions [10,18], we enhanced the real-time performance obtaining a total latency of about 140 ms, defined as the sum of the processing time ( 10 ms, RHS configuration) and the ATC widow ( 130 ms). Again, since the relevant upgrade in our architecture was to move towards an embedded device (i.e., Raspberry Pi), we confirm how the lightness and low-complexity of the ATC technique perfectly match with the low-processing capability of an embedded system, while maintaining adequate FES control, usability and power performance.

## 6. Conclusion and Future Perspectives

In this paper we presented our last prototype of the sEMG(ATC)-controlled-FES system, in which we have been replaced the previous MATLAB^®^ & SIMULINK^®^ software architecture [10] with the novel embedded version running on a Raspberry Pi, in order to overcome the performances limitation due to the use of a general purpose computer. Taking the advantages of the object-oriented and multi-threaded approach, along with the versatility of Python programming language, we developed a multi-platform software core able to work onto several devices and with different operating systems, also enhancing system usability by featuring a graphical user interface.

Since the main tasks of the system application concern the modulation of the FES pattern and its real-time application, we designed a processing structure able to match with the low computational power of an embedded device. We implemented a calibrated ATC-FES lookup table structure, combined with noise-gateway controls, which allows us to obtain a total processing time, defined as the delay between ATC data and the new FES parameters, below 30 ms (corner cases), obtained without substantally impact on the CPU and RAM usage, therefore demonstrating the lightness and responsiveness of the event-driven technique in the control of the stimulation.

We proved system efficiency by studying the similarity between voluntary and stimulated movements in therapist-patient real FES scenarios (healthy subjects): using the maximum of the normalized cross correlation coefficient, as comparison measurement between the signals of the involved limbs, we obtained a mean value of 0.87 ± 0.07 as result of 60 repetitions (5 therapist-patient couples per 12 repetition each one) during the reproduction of the elbow flexion movement. These promising outcomes allowed us also to preliminarily evaluate the FES performance for the knee extension movement: Adopting the same methodologies, we analyzed 13 exercise repetitions achieving a correlation value of 0.93 ± 0.02.

Future improvements, i.e., full FES parameters modulation, pre-FES movement recognition, will permit us to further optimize the ATC-based stimulation in order to extend our testing phase to a wide list of rehabilitation exercises, also performing some clinical trials with the support of medical staff.

## Figures and Tables

**Table 1:** Tested hardware configurations. Main differences concern the acquisition device (single channel or four-channel board) and the control unit (GNU/Linux Raspberry or Microsoft^®^ Windows^®^ PC).

	Acquisition Device		Control Unit		BLE Module
Config.	Single Channel	4-Channel Board	GNU/Linux Raspberry	Microsoft^®^ Windows^®^ PC	CC2540 *
**C1**	✓		✓		
**C2**	✓		✓		✓
**C3**		✓	✓		✓
**C4**	✓			✓	✓
**C5**		✓		✓	✓

* up to three concomitant connections.

**Table 2:** Time profiling results for the evaluation of the methods breakdown during the FES current definition process.

	Configuration				
Method	C1	C2	C3	C4	C5
queue	90.71%	92.78%	88.03%	97.41%	97.25%
append	1.43%	1.03%	2.35%	-	-
median	1.65%	1.40%	2.24%	0.53%	0.52%
FES_start	5.11%	3.81%	6.04%	1.57%	1.63%
plot	0.68%	0.62%	0.81%	-	-
	**100%**	**100%**	**100%**	**100%**	**100%**

**Table 3:** Time profiling results for the Plotting process into its two sub-threads: get_value and sleep methods represent the active and inactive action, respectively, of the clock object which manages the plotting of the data.

	Configuration				
Method	C1	C2	C3	C4	C5
get_value	0.94%	0.93%	0.95%	0.45%	0.41%
sleep	99.06%	99.07%	99.05%	99.55%	99.59%
	**100%**	**100%**	**100%**	**100%**	**100%**

**Table 4:** CPU and RAM measurements of the application main stages, which have been evaluated testing the Reference Hardware Setup (RHS) with four working channels.

Stages	CPU (%)	RAM (MB)
Login	20	84
Initialization	21	85
Threshold calibration *	24.1	88.9
ATC maximum calibration *	26	87
AROM evaluation *	32	89
Maximum current calibration *	45.7	89
Stimulation	73.2	87.8
Parameters	15	88.3

* four steps of the Calibration process.

**Table 5:** RHS resources performance during the Stimulation stage depending on the number of working channels and the FES current definition implementation. The ATC processing has been enabled (standard flow) or disabled (direct ATC-I_FES_ equivalence) in order to study if whether implementation affects the run-time system performance.

		1 Ch.	4 Ch.	1 Ch.	4 Ch.
**Process**	I/O operations	✓	✓	✓	✓
	ATC processing	✓	✓	x	x
**Resources**	CPU (%)	53.8	73.2	53	74.4
	RAM (MB)	87.7	87.8	91	92

**Table 6:** sEMG-trigger-FES systems table comparison.

Work	Control Feature	FES Parameter	Processing HW	Embedded	Wireless	Modular System	#Ch	Latency (ms)
[49]	RMS	intensity	MCU	✓	Bluetooth	x	8	300
[50]	envelope	intensity	n.a.	x	x	n.a.	4	n.a.
[51]	threshold crossing	frequency	MCU	✓	335/433 MHz	x	2	142
[52]	force angle	intensity	PC	x	x	n.a.	4	n.a.
[53]	sEMG IMU	intensity width	MCU	✓	Bluetooth 2.1	x	4	21
[54]	envelope	on/off stimuli	PC	x	x	x	1	1600
[55]	entropy	frequency width	MCU	✓	x	x	1	300 ^1^
[10,18]	ATC	intensity	PC	x	Bluetooth 4.2	✓	4	774.5 ^1^ 932 ^1^
**This**	ATC	intensity	Raspberry	✓	Bluetooth 4.2	✓	4	140

^1^ measured as therapist-patient delay.
